# Gastric Cancer, Immunotherapy, and Nutrition: The Role of Microbiota

**DOI:** 10.3390/pathogens13050357

**Published:** 2024-04-26

**Authors:** Pauline Raoul, Valeria De Gaetano, Gianmario Sciaraffia, Ginevra Ormea, Marco Cintoni, Carmelo Pozzo, Antonia Strippoli, Antonio Gasbarrini, Maria Cristina Mele, Emanuele Rinninella

**Affiliations:** 1 Clinical Nutrition Unit, Department of Medical and Abdominal Surgery and Endocrine-Metabolic Sciences, Fondazione Policlinico Universitario Agostino Gemelli IRCCS, 00168 Rome, Italymarco.cintoni@unicatt.it (M.C.); mariacristina.mele@unicatt.it (M.C.M.); 2 School of Specialization in Internal Medicine, Catholic University of the Sacred Heart, 00168 Rome, Italy; valeria.degaetano01@gmail.com (V.D.G.); gianmario.sciaraffia@gmail.com (G.S.); 3 Degree Course in Pharmacy, Catholic University of the Sacred Heart, 00168 Rome, Italy; ginevra.ormea01@icatt.it; 4 Research and Training Center in Human Nutrition, Catholic University of the Sacred Heart, 00168 Rome, Italy; antonio.gasbarrini@unicatt.it; 5 Comprehensive Cancer Center, Fondazione Policlinico Universitario Agostino Gemelli IRCCS, 00168 Rome, Italy; carmelo.pozzo@policlinicogemelli.it (C.P.); antonia.strippoli@policlinicogemelli.it (A.S.); 6 Digestive Disease Center (CEMAD), Department of Medical and Abdominal Surgery and Endocrine-Metabolic Sciences, Fondazione Policlinico Universitario Agostino Gemelli IRCCS, 00168 Rome, Italy; 7 Department of Translational Medicine and Surgery, Catholic University of the Sacred Heart, 00168 Rome, Italy

**Keywords:** gastric cancer, immunotherapy, ICI, nutrition, diet, gut microbiota, *H. pylori*, nutritional status, immunotherapy response, muscle mass, malnutrition

## Abstract

Immune checkpoint inhibitors (ICI) have revolutionized the treatment of gastric cancer (GC), which still represents the third leading cause of cancer-related death in Western countries. However, ICI treatment outcomes vary between individuals and need to be optimized. Recent studies have shown that gut microbiota could represent a key influencer of immunotherapy responses. At the same time, the nutritional status and diet of GC patients are also predictive of immunotherapy treatment response and survival outcomes. The objective of this narrative review is to gather recent findings about the complex relationships between the oral, gastric, and gut bacterial communities, dietary factors/nutritional parameters, and immunotherapy responses. Perigastric/gut microbiota compositions/functions and their metabolites could be predictive of response to immunotherapy in GC patients and even overall survival. At the same time, the strong influence of diet on the composition of the microbiota could have consequences on immunotherapy responses through the impact of muscle mass in GC patients during immunotherapy. Future studies are needed to define more precisely the dietary factors, such as adequate daily intake of prebiotics, that could counteract the dysbiosis of the GC microbiota and the impaired nutritional status, improving the clinical outcomes of GC patients during immunotherapy.

## 1. Introduction

Gastric cancer (GC) is the third leading cause of cancer-related death [1]. In Western countries, where screening for GC is not routinely performed, the diagnosis often occurs at advanced stages. Symptoms of underlying GC generally include weight loss, dysphagia, dyspepsia, vomiting, early satiety, and/or iron deficiency anemia [2]. Once the diagnosis and staging of GC are performed, multidisciplinary treatment planning is mandatory. In its early stages, surgical resection might be potentially curative. Patients with inoperable, locally advanced, and/or metastatic disease should be considered for systemic treatment [2]. Commonly used therapies include chemotherapy, radiotherapy, and targeted therapy. In this scene, immune checkpoint inhibitors (ICI) have revolutionized the treatment of GC. However, the overall survival rates widely vary between individuals after more than 12 weeks of ICI treatment [3]. Consequently, new biomarkers are needed to better select patients for this type of cancer therapy and develop therapeutic strategies to optimize survival outcomes. Considering that ICI, by definition, controls the host immune system, recent studies [4,5] have demonstrated the close interactions between the gut microbiome and the host immune system, especially in GC. Some bacterial species, such as *Roseburia*, *Bifidobacteria*, and *Faecalibacteria*, may produce beneficial metabolites such as short-chain fatty acids (SCFAs), which can counteract inflammation and tumorigenesis pathways, while bacterial species can produce metabolites such as specific secondary bile acids—such as deoxycholic acid and lithocholic acid—and toxins that potentiate the carcinogenesis process, inducing DNA damage and genomic instability [6]. A recent study has shown that gut microbiota is associated with clinical response to anti-programmed cell death protein 1 (PD-1)/ programmed cell death ligand 1 (PD-L1) immunotherapy in gastrointestinal cancers and could consequently be potential predictive biomarkers of response [7]. At the same time, dietary habits and nutritional status may play an important role in affecting GC development and particularly immunotherapy responses through gut microbiota modulation [8]. Thus, during the GC course, the microbiota may be shaped by dietary interventions impacting gut inflammation, the mucosal immune response, and the synthesis or modulation of oncologic molecular processes. This review aims to highlight the potential relationships between nutrition, microbiota, and immunotherapy outcomes in GC to develop dietary strategies that could counteract dysbiosis and improve clinical results.

## 2. Gastric Cancer and Microbiome

### 2.1. Perigastric Microbiota in GC

#### 2.1.1. The Role of *Helicobacter pylori*

*H. pylori* is a Gram-negative bacterium that colonizes the epithelial cells that compose the gastric epithelium. Its infection is often asymptomatic but can lead to several gastric diseases, including peptic ulcer disease (PUD), chronic gastritis, mucosa-associated lymphoid tissue lymphoma (MALT), and GC [9]. The International Agency for Research on Cancer has categorized it as a class I carcinogen. *H. pylori* infection affects about 50% of the world’s population and generally induces chronic inflammation of the gastric mucosa, of which 5 to 15% evolve in gastric and duodenal ulcers and less than 1% in GC. This different behavior is correlated to the heterogeneity of the bacterial genome and its different virulence factors. “Correa’s cascade” has proved that intestinal-type GC originated from *H. pylori*-induced chronic gastritis, followed by gastric atrophy, intestinal metaplasia, and then dysplasia [10]. The first part of carcinogenesis is highly dependent on *H. pylori* because the inflammatory process is related to the presence of bacterial virulence factors, but the next stages are *H. pylori*-independent. A study has demonstrated that colonization levels decrease in subjects with metaplasia and dysplasia and disappear in the adenocarcinoma stage [11]. More precisely, microbial alpha diversity (within sample diversity) and beta diversity (diversity between samples) were assessed in patients with GC and patients with chronic gastritis [11]. Researchers found that microbial diversity significantly decreased in GC patients compared with patients with chronic gastritis (*p*-value = 0.003) [11].

*H. pylori* plays a crucial role in GC development, acting directly through its virulence factors but also indirectly causing an alteration of microbiota composition. The major virulent factor is the cytotoxin-associated gene A (CagA) protein, which is secreted by the type IV secretion system in the cytoplasm of gastric epithelial cells. It can promote cell proliferation thanks to its interaction with different signaling pathways such as phosphoinositide 3-kinase (PI3K), alpha serine/threonine kinase (AKT), WNT, and nuclear factor kappa (NFKB) [12], and it can reduce epithelial cell apoptosis by inhibiting tumor protein 53 TP53 [13]. *H. pylori* infection and inflammation cause gastric mucosal barrier disruption due to an alteration of tight junctions and epithelial–mesenchymal transition (EMT) in gastric epithelial cells, which promotes the invasion and proliferation of cancer cells [14]. *H. pylori* can cause gastric mucosal barrier disruption, altering tight junctions and EMT. Its infection and the resulting inflammation stimulate the proliferation of cancer cells [15]. Moreover, this bacterium can suppress antitumor immunity and protect GC cells from immune responses, increasing PD-L1 expression in gastric epithelial cells. This is one of the main immune tolerance mechanisms that prevents T cells from attacking malignant cells [16]. It has been demonstrated that *H. pylori* infection can greatly change the alpha diversity of the gastric microbiota [17]. It maintains an inflammatory response, which leads to the loss of acid-secreting parietal cells and an increase in gastric pH.

A recent cross-sectional study has also shown that *H. pylori* colonization progressively decreases, so other bacteria can colonize the gastric mucosa, resulting in dysbiosis [18]. Indeed, Miftahussurur et al. analyzed the 16S ribonucleic acid (RNA) of more than 130 gastric biopsy specimens: 27 were *H. pylori*-positive and 110 were *H. pylori*-negative. Significantly lower α-diversity was found in *H. pylori*-infected patients compared with noninfected patients (all *p*-values < 0.001). This imbalance and the continuous stimulation of the host immune system are triggered by the chronic inflammation of the gastric mucosa and tumor carcinogenesis [19].

#### 2.1.2. Other Perigastric Bacteria Involved in GC

Actinobacteria, Bacteroidetes, and Firmicutes are the main phyla that make up the gastric microbiota of *H. pylori-negative* subjects [20]. Several studies have demonstrated that *H. pylori* colonization will lead to dysbiosis and an increase in other bacteria, such as Spirochetes and Proteobacteria. Dysbiosis is a dynamic process that correlates with cancer progression. Indeed, several studies have confirmed significant differences in microbiota composition balance in patients with chronic (atrophic) gastritis, metaplasia, and GC [21]. Microbiota profiles in patients with *H. pylori-induced* mucosal gastritis or glandular atrophy are dominated by *Helicobacter* and, minorly, by *Streptococcus*, *Prevotella*, and *Neisseria*. Therefore, this results in a reduction in microbiota richness, diversity, and evenness compared with patients with typical gastric mucosa [22]. The loss of glandular tissue and the decreased acid secretion that occur in GC cause a reduction in *H. pylori* invasion and the enrichment of intestinal commensals, such as *Lactobacillus*, *Enterococci*, *Carnobacterium*, *Parvimonas*, *Citrobacter*, *Clostridium*, *Achromobacter*, and *Rhodococcus* [11], and oral microbiota, such as *Fusobacterium nucleatum*, *Veillonella*, *Leptotrichia*, *Haemophilus*, and *Campylobacter* [23]. Some species, such as *Fusobacterium nucleatum*, are associated with a worse prognosis in diffuse-type GC. These bacteria can produce toxins that promote the progression of GC. Through different types of toxins and superoxides, they induce DNA damage, genomic instability, and epigenetic changes [24].

Microbes with nitrosating capability (especially *Staphylococcus*, *Lactobacillus*, and *Escherichia coli*) play a crucial role because they produce N-nitroso compounds and polyamines that can suppress antitumor immunity and influence cell proliferation, invasion, and metastasis [25]. It has been demonstrated that chronic therapy with H2 receptor antagonists (H2RA) or proton pump inhibitors, used for gastrointestinal disorders including erosive esophagitis and gastroesophageal reflux disease (GERD), can raise the colonization of nitrosating bacteria and the transformation of nitrogen compounds into N-Nitroso carcinogens [26]. The decreased gastric acidity promotes the risk of bacterial overgrowth and alters the community of gastric microbiota [27].

##### *Staphylococcus* 

Urease-positive *Staphylococcus epidermidis* (*S. epidermidis*) was frequently isolated from gastric biopsy specimens. Two strains of *S. epidermidis* inhibited the growth of all *H. pylori* strains. In a recent study, *S. epidermidis* was inoculated into germ-free mice infected with *H. pylori* to ascertain whether these bacteria could influence *H. pylori*-associated pathogenesis [28,29]. *S. epidermidis* coinfection with *H. pylori* did not significantly change stomach clinical conditions, but the proinflammatory cytokine gene levels such as interleukin (Il)-1β and Il-22 were significantly lower compared with *H. pylori*-monoinfected mice.

Moreover, a recently published paper [30] described the gastric microbiome in *H. pylori*-infected individuals living in two Colombian populations with high (Tumaco) and low (Túquerres) GC risk. Gastric microbiota analyses of 20 individuals were performed. Multiple operational taxonomic units (OTUs) were detected exclusively in either group. Two OTUs, *Veillonella* spp. and *Leptotrichia wadei*, were significantly more abundant in Túquerres, while more than 15 OTUs, including *Staphylococcus* spp. and *S. epidermidis*, were significantly more abundant in the other populations from Tumaco.

##### *Lactobacillus* 

The Lactobacillaceae family acts as a carcinogenic factor by producing lactic acid, which may serve as an energy source for tumor cells and stimulate tumor angiogenesis [31]. Lactic acid could create an immunosuppressive tumor microenvironment. Indeed, it enhances the production of vascular endothelial growth factor and arginase 1 and mediates M2-like polarization of tumor-associated macrophages. Lactic acid inhibits T-cell and natural killer cell function and increases the number of myeloid-derived suppressor cells, which can further suppress natural killer cell cytotoxicity [32]. The Lactobacillaceae family can also enhance the effect of *H. pylori* on human monocyte-derived dendritic cells, leading to dendritic cell maturation and induction, exacerbating the *H. pylori*-mediated inflammatory response, and promoting gastric carcinogenesis [33].

##### *Fusobacterium nucleatum* 

It has been reported that *Fusobacterium nucleatum* is more abundant in the microbiota of GC patients than in non-tumor controls [34]. *Fusobacterium nucleatum* has a direct interaction with epithelial cells by invading them with FadA adhesion molecules that bind E-cadherin on the cell surface and by activating Wnt [35]. Its carcinogenic mechanism is not fully understood, but it impacts metabolic function, dysregulation of actin dynamics, and cancer cell motility. *Fusobacterium nucleatum* could activate the nuclear factor-kappa B (NF-κB) pathway to stimulate the production and release of inflammatory cytokines, such as IL-1β, IL-6, IL-8, and tumor necrosis factor (TNF), thereby creating a proinflammatory microenvironment that favors tumor development [36,37]. Moreover, the adhesion of *Fusobacterium nucleatum* from FadA to the E-cadherin of intestinal epithelial cells drives the activation of the Wnt/β-catenin pathway to promote the proliferation of tumor cells [35].

### 2.2. Gut Microbiota in GC

The gut microbiota is significantly different in terms of composition according to GC types [38,39,40]. The abundance of specific bacteria could increase due to increased nucleotide metabolism and nitrogen-containing compounds [41,42]. A recent study found that in fecal samples of GC patients, *Desulfovibrio* was more prevalent compared with healthy patients, thereby contributing to inflammation capable of inducing carcinogenesis [40]. Furthermore, the composition of the cancer gut microbiota differs according to human single nucleotide polymorphisms among patients from different geographical areas [43]. In Asia, Firmicutes is the most dominant phylum, whereas in Europe, Proteobacteria is [43].

GC treatments can also accentuate gut cancer microbiota dysbiosis. Indeed, fecal samples of patients after gastrectomy are more abundant in the quantity of *Escherichia coli*, *Enterobacter*, and *Streptococcus* [39,44]. Bacterial species diversity and richness, as well as the abundance of *Streptococcus*, *Veillonella*, and *Atopobium*, increase after gastrectomy [39]. As regards other treatments, such as chemotherapy, the differences in terms of the composition of the gut microbiota remain to be studied.

Furthermore, although the mechanisms of the relationships between *H. pylori* presence and non-*H. pylori* bacteria remain unclear, the infection of *H. pylori* could be associated with an increase in levels of *Proteobacteria*, *Spirochetes*, *Akkermansia*, and *Acidobacteria* and a depletion in the abundance of the phyla Actinobacteria, Bacteroidetes, and Firmicutes [45,46,47].

It has also been shown that gastrointestinal cancer can be caused by a dysregulation of the expression of non-coding RNA (ncRNAs’) through the gut microbiome. Indeed, abnormalities in the expression of mRNAs can result in pathological processes that contribute to the onset and spread of cancer. Yuan et al. showed that tumoral tissues exhibit higher expression levels of miR-17-92, miR-21, and miR-503 compared with normal tissues [48]. The expression of RAS p21 and programmed cell death-4 is downregulated by MiR-21. In particular, in colorectal cancer (CRC), *Fusobacterium nucleatum* infection upregulates the expression of miR-21 while downregulating miR-18a and miR-4802 [49]. In the gut, microRNA-microbiota crosstalk is essential for maintaining gut homeostasis. Dietary or secreted microRNAs from intestinal epithelial cells can affect the composition of the microbiota. The amount of miRNA released and the microbiota are regulated in both directions [50]. The gut microbiome and its pathway mechanisms are still not fully known. Understanding these pathways is the key to improving treatment options.

## 3. Gastric Cancer and Immunotherapy

### 3.1. The Rationale behind Immunotherapy: The Tumor Microenvironment

The tumor microenvironment (TME) is the result of a highly complex and dynamic interplay between tumor cells and stroma, immune cells, and cytokines. In the last few years, the TME and its role in response to therapies or the progression of GC have become an issue of relevance. In particular, studies on profiling immune infiltration have shown that the abundance and type of immune cells (innate and adoptive) in TME significantly influence immunotherapy [51]. Following the theory of the cancer-immunity cycle, when cancer cells die, released antigens are captured by dendritic cells (DCs) and presented to the effector T (Teff) cells to respond against the cancer-specific antigens. Once activated, Teff cells infiltrate the tumor bed, bind to cancer cells, and drive apoptosis. In cancer patients, this process is suppressed by some immune rheostat factors (such as PD-L1/PD-1) present in the microenvironment [52]. Usually, TME is classified into two phenotypes: non-inflamed (cold tumors) and immune-inflamed (hot tumors), according to the infiltration of immune cells [53]. Hot tumors can activate immune-function-related pathways, while cold tumors are characterized by genomic instability and transcriptional changes that may promote mutagenesis. A study on the expression of CD3, CD4, CD8, CD45RO, and FOXP3 in randomly selected resected gastric adenocarcinoma specimens from 88 North American patients showed that the expression of immune cell density was independent of anatomic staging. At the invasive margin, high expression of CD3, CD4, CD8, and CD45RO, along with CD4 and FOXP3, at the tumor center was associated with improved overall survival [54]. In mouse models, cytotoxic CD8+ lymphocytes have been observed to be responsible for tumor cell death and apoptosis by direct antigen recognition and inducing rejection; the presence of this subpopulation of lymphocytes may reflect a robust immune response to tumor antigens, resulting in better survival [55].

On the other hand, the role of CD4^+^ helper T cells is less understood. While the interaction with the major histocompatibility complex (MHC II) may induce macrophages and CD8+ activation against tumor cells, it may also lead to differentiation into regulatory T cells (such as FOXP3+ cells) and inhibit the immune response [56]. As previously indicated in the study of Uppal et al. [54], there is intratumoral heterogeneity between the tumor center and invasive margin. This is evident in the observation of FOXP3+ cell expression. Indeed, while the presence of FOXP3+ cells at the tumor center has been associated with better survival, the expression of FOXP3+ cells at the invasive margin is correlated with a worse prognosis. These findings may suggest that regulatory T cells in the tumor center are an expression of an established immune response; on the other hand, at the invasive margin, it may indicate an immune system’s inability to identify tumor cells as pathologic.

CD45RO^+^ regulatory T cells, derived from CD4^+^ cells, function as memory cells; high expression of this subpopulation is a consequence of an effective immune response and correlates with better survival [57]. Ren et al. [53] studied the correlation between the proportion and infiltration of immune and stromal cells in GC and the pathological characteristics and clinical outcomes. They found that elevated levels of Tregs, CD8^+^ Tems, platelets, and sebocytes and decreased levels of fibroblasts were more frequently present in older patients. Notably, CD8^+^ T cells and memory B cells were increased in patients with a higher T stage, grade, and advanced pathological stage. In addition, microsatellite instability was strongly associated with the presence of Th1, Th2, pro-B, NK and endothelial cells, basophils, and Tregs. This is probably due to the larger number of mutated genes that result in more mutation-related neoantigens, which activate a stronger immune response. Interestingly, gender was found to have a role; in fact, male patients showed higher levels of immune effector cells (CD4^+^ T cells, CD4^+^ memory T cells, and CD8^+^). Results showed that pro-B, Th1, megakaryocyte–erythroid progenitor, NK, Treg, and CD4^+^ memory T cells were associated with favorable OS and PFS, while stromal cells correlated with poor prognosis, as they might affect the infiltration of immune cells [58]. The classification of gastric adenocarcinoma has been proposed based on molecular profiling: (i) Tumors with chromosomal instability, characterized by aneuploidy and focal amplification of receptor tyrosine kinases; (ii) genomically stable (GS) tumors, which are enriched for the diffuse histological variant and mutations of RHOA or fusions involving RHO-family GTPase-activating proteins; (iii) Epstein–Barr Virus positive (EBV^+^) GC, with recurrent tPIK3CA mutations, extreme DNA hypermethylation, and amplification of JAK2, PD-L1, and PD-L2; (iv) microsatellite unstable tumors, which showed elevated mutation rates. EBV+ GC and MSI GC have rich lymphocytic infiltration in the tumor stroma, are rich in CD8 T cells, and are capable of mounting a robust antitumor inflammatory response [59]. On the other hand, in the major expression of PD-L1 expression, a more significant increase in the number of CD8^+^ T cells at the tumor-invasive front has been observed [60]. In a post hoc analysis of the ITACA-S trial (sequential chemotherapy regimen vs. 5-FU/LV monotherapy as adjuvant treatment in patients with radically resected GC), microsatellite instability (MSI) was found to have a positive prognostic value with better disease-free survival and overall survival; even inflammatory reactions were related to better overall survival, prognostic, and predictive value of MSI, inflammatory reaction, and PD-L1 in GC. Nowadays, the relationship between the expression of PD-L1 and prognosis is still controversial.

A systematic review highlighted that EBV infection and MSI were found to be associated with the expression of PD-L1, and its overexpression was a significant adverse prognostic factor [61]. Focusing on clinical pathological features, PD-L1 overexpression was related to deeper tumor infiltration, positive lymph node metastasis, and positive venous invasion, suggesting that this subgroup of patients would benefit from treatment with ICI. On the other hand, no clear relationship with sex, age, tumor site, tumor size, tumor differentiation, Lauren classification, TNM stage, lymphatic invasion, or neural invasion was observed. Another systematic review, including eleven studies with 2298 patients, showed no prognostic effect of PD-L1 and TILs in GC patients [61].

### 3.2. Immunotherapy Options for GC: A State-of-the-Art

During the last ten years, immunotherapy has quickly gained attention due to its antitumor efficacy [62]. Particularly, ICIs play an active role in the treatment of GC. In Europe, for advanced esophagogastric adenocarcinoma, first-line therapy in Her-2-negative tumors includes (i) nivolumab in association with chemotherapy in patients with PD-L1 CPS (combined positive score) ≥ 5 [63]; (ii) pembrolizumab plus chemotherapy in PD-L1 CPS ≥ 10; (iii) doublet or triplet chemotherapy alone in PD-L1 CPS < 5 [64]. The PD-L1 expression was analyzed using the Combined Positive Score (CPS). In the case of progression disease, second-line therapy is approved for the association of ramucirumab and chemotherapy, while third-line therapy accounts for trifluridin/tipiracil or best supportive care [65]. As a third-line therapy, monotherapy with pembrolizumab received approval in the USA after the KEYNOTE-059 study in patients with CPS ≥ 1. Furthermore, the association of pembrolizumab + trastuzumab + chemotherapy has been approved as first-line treatment in advanced Her2 positive tumors in the USA (KEYNOTE-811) and is expected to be approved in Europe in 2024; at the moment, patients with expression of Her2 undergo trastuzumab plus chemotherapy as first-line therapy, while in progress disease, the new standard is trastuzumab/deruxtecan. The DESTINY-Gastric02 study shows clinically meaningful activity of trastuzumab deruxtecan, with durable responses in patients with Her2-positive gastric or gastro-oesophageal junction adenocarcinoma whose disease progressed on a trastuzumab-containing regimen [66]. The later line is monotherapy with docetaxel/irinotecan/paclitaxel/ramucirumab is performed.

While already present in first-line treatment of advanced GC, the role of checkpoint inhibition in perioperative treatment is under investigation in several trials. In the randomized phase II DANTE trial of the Association of Medical Oncology of the German Cancer Society (AIO), atezolizumab (anti-PD-L1) plus chemotherapy was compared to the standard monochemotherapy in patients with resectable EGC. The first results of safety show a safe application of the combination, while the first efficacy results are expected [67]. The KEYNOTE-585 trial investigates the administration of pembrolizumab plus chemotherapy vs. chemotherapy + placebo in the postoperative setting. Indeed, a benefit to overall survival from the combination of durvalumab with FLOT in a perioperative setting is investigated within the MATTERHORN trial, a global double-blind placebo-controlled phase III trial [68].

Despite its promising role in more lines of treatment, the efficacy of immunotherapy is still affected by primary resistance, observed in 60–70% of cases. It is due to low mutational burden, poor intrinsic antigenicity of tumor cells [69], absence of priming by potentially immunogenic pretreatment with chemotherapy or radiotherapy [70], defective antigen presentation during the priming phase [71], local immunosuppression by extracellular metabolites [72], and functional exhaustion of tumor-infiltrating lymphocytes [72,73].

Nowadays, immunotherapies against advanced GC include ICIs, adoptive cell therapy, cancer vaccines, vascular endothelial growth factor A (VEGFA), and antibody anti-chimeric antigen receptor (CAR-T).

#### 3.2.1. Immune Checkpoint Inhibitors

Immune checkpoint inhibitors account for anti-PD-1, anti-PD-L1, and anti-CTLA4 antibodies. In general, the immune system modulates its response through the expression of PD-1 on activated immune cells, which binds its ligand (PD-L1), resulting in immune cell apoptosis and immune suppression. In advanced GC, PD-L1 is overexpressed, leading to the evasion of tumor cells and the progression of cancer. Anti-PD-1 antibodies are nivolumab, pembrolizumab, sintilimab, tislelizumab, retifanlimab, and tebotelimab. In particular, nivolumab has been approved to treat advanced and recurrent GC [74], while pembrolizumab has shown good efficacy and moderate side effects in the KEYNOTE-158 study, a phase II trial among advanced GC [75]. Anti-PD-L1 antibodies, including atezolizumab, avelumab, durvalumab, retifanlimab, and tebotelimab, were also approved for the treatment of multiple advanced cancers. CTLA-4 indeed inhibits CD28 signaling, a critical pathway in T cell activation, interacting with high affinity with B7-1/B7-2 [76]. The combination of ipilimumab and nivolumab has been approved to treat advanced GC, even though the efficacy of CTLA-4 inhibitors as a monotherapy needs further investigation. The Food and Drug Administration approved a new indication for dostarlimab, a PD-1 receptor-blocking antibody, for the treatment of adult patients with mismatch repair-deficient recurrent or advanced solid tumors who have progressed on or following prior treatment and who have no satisfactory alternative treatment options. This indication received accelerated approval based on tumor response rate and durability of response [76].

#### 3.2.2. Anti-Angiogenic Therapy

VEGFA plays an essential role in angiogenesis, modulates the immune response, resulting in the escape of tumor cells from surveillance, and can promote the transfer of Tregs to tumor sites [48]. Several phase 3 studies showed that anti-angiogenetic agents, like ramucirumab, are active in later lines of therapies, i.e., in pretreated metastatic GC patients alone or in conjunction with chemotherapy. Dual inhibition of the VEGF–VEGFR2 and PD-1–PD-L1 (pembrolizumab plus ramucirumab) pathways has been investigated in a multicohort, non-randomized, open-label, phase 1a/b trial and showed a manageable safety profile with favorable antitumor activity in patients with previously treated advanced gastric or gastroesophageal junction adenocarcinoma [77].

#### 3.2.3. Adoptive Cell Therapy

Adoptive cell therapy can induce an effective immune response through the extraction of in vivo T lymphocytes (such as tumor-infiltrating lymphocytes, lymphokine-activated cells, and cytokine-induced killer cells), selection for or introduction of tumor-reactive cells, in vitro expansion, and delivery of the T-cell product back to the patient [78]. In patients with melanoma, previous treatment with chemotherapeutic agents or total body irradiation is performed to obtain lymphodepletion and augment the functions of adoptively transferred T cells. Mechanisms underlying this strategy include (a) the depletion of regulatory T cells and myeloid-derived suppressor cells that limit the function and proliferation of adoptively transferred cells; (b) the removal of immune cells that act as “sinks” for homeostatic cytokines, whose levels increase after lymphodepletion; and (c) the activation of the innate immune system via Toll-like receptor 4 signaling, which is engaged by microbial lipopolysaccharide that translocated across the radiation-injured gut [79].

#### 3.2.4. Cancer Vaccines

Cancer vaccines are another form of immunotherapy targeting antigens that are expressed only by cancer cells [80]. Cancer vaccines could be cellular vaccines, protein/peptide vaccines, or genetic vaccines [81]. In the last few years, in GC, various trials have focused on cancer vaccines, but, up to date, the results remain inconclusive. Indeed, tumor-associated antigens have been targeted by vaccines with limited efficacy, even in the case of combined chemotherapy or radiotherapy [82,83,84]. Small extracellular vesicles can also be studied as cancer vaccines; they are nanoscale vesicle structures secreted by almost all cells that can transmit information between cells and participate in their physiological and pathological processes. In particular, they showed a role in the initiation, progression, angiogenesis, metastasis, and chemoresistance of GC [67]. With the use of nanotechnology, engineered exosomes are emerging as new cancer vaccines [85].

#### 3.2.5. CAR-T Cell Therapy

Chimeric antigen receptor (CAR) T cells are composed of an extracellular single-chain variable fragment (scFv) that recognizes diverse tumor-associated antigens and activates the phosphorylation cascade in T cells, leading to the release of cytotoxic granules, the transcription of genes encoding cytokines, and cell proliferation [86]. Although CAR-T therapy has shown an antitumor effect in advanced GC, it is not immune from threatening side effects related to release syndrome and CAR-T therapy-related encephalopathy (chills, fever, nausea, neurotoxicity, and cardiotoxicity).

## 4. The Influence of the Gut Microbiota on GC Immunotherapy Responses

It is widely known that the gut microbiota plays an essential role in local and systemic immune responses. In recent years, researchers have focused their attention on the effects of the microbiota and its metabolites on the cancer-immune system and immune checkpoint inhibitor therapeutic response. Preclinical studies on mice have shown that gut microbiota could stimulate CD8^+^ T cells and Th1 activation by commensal bacteria, influencing cancer responses [87], while exposure to antibiotics could attenuate the effects of cancer therapy [88]. Human and animal studies highlighted the role of gut microbiota by demonstrating that composition and diversity were predictive of response to ICI immunotherapy in melanoma patients; furthermore, patients with advanced non-small cell lung cancer (NSCLC), renal cell carcinoma (RCC), and urothelial carcinoma treated with PD-1/PD-L1 mAb who underwent antibiotic treatment showed significantly shorter progression-free survival (PFS) and overall survival [89].

Hannani et al. demonstrated that the antitumor effects of cytotoxic T lymphocyte-associated protein 4 (CTLA-4) blockade may depend on distinct Bacteroides species. As observed both in mice and patients, the efficacy of CTLA-4 blockade was markedly curtailed in antibiotic-treated mice and was restored by gavage with *Bacteroides fragilis* (*B. fragilis*), by immunization with *B. fragilis* polysaccharides or by adoptive transfer of *B. fragilis*–specific T cells. They also assessed the efficacy of fecal microbial transplantation by confirming that treatment of melanoma patients with antibodies against CTLA-4 favored the outgrowth of *B. fragilis* with anticancer properties [90].

Sivan et al. compared melanoma growth in mice harboring distinct commensal microbiota. They demonstrated that the differences in spontaneous antitumor immunity were eliminated upon cohousing or after fecal transfer. They also found that *Bifidobacterium* was associated with the antitumor effects. Furthermore, after oral administration of *Bifidobacterium*, they observed an improvement in tumor control to the same degree as PD-L1-specific antibody therapy (checkpoint blockade), and combination treatment nearly abolished tumor outgrowth, probably due to an augmentation of dendritic cell function and an enhancement of CD8^+^ T cells [91].

In 2018, the analysis of melanoma fecal microbiome samples from 112 patients showed significantly higher alpha diversity and relative abundance of *Ruminococcaceae* bacteria in responding patients treated with anti-PD-1 immunotherapy [92]. In 2021, in patients affected by melanoma refractory to ICIs, fecal microbiota transplantation resulted in the ability to promote response to anti-PD-1 immunotherapy [93]. The gut microbiota can reprogram the tumor microenvironment by acting on both innate and adaptive immunity; it can elicit intermediate effects of immune cells and antitumor effects of adaptive immune cells; it can also increase the immunogenicity of tumor cells, as well as provide an alternative source of energy or immune cells [94].

After total body irradiation, microbiota translocation from the gut induces activation of DCs to express proinflammatory cytokines and promotes the effects of adoptive T-cell therapy [87]. Antibiotics inhibit microbial translocation, decreasing the number of activated DCs and thereby impairing antitumor T cell-mediated immunity [95].

Furthermore, high microbial diversity in the gastrointestinal tract has been associated with increased efficacy of ICI treatment [89,93]. The gut microbiota may influence the toxicity of ICIs [96]. Certain microbes increase ICI-induced toxicity, whereas others decrease the risk of immune-mediated adverse events, such as *B. fragilis* and *Burkholderia cepacia* for CTLA-4-induced colitis [97]. However, the identities of specific microbes with protective or adverse effects on ICI-induced toxicity remain uninvestigated.

As far as GC is concerned, *H. pylori* is widely known as a major risk factor for progression from chronic superficial gastritis to atrophic gastritis, intestinal metaplasia, dysplasia, and eventually to GC. It is also responsible for systemic immunomodulation and is related to the inefficacy of immunotherapy in cancers such as melanoma and NSCLC [98]. Che et al. demonstrated that *H. pylori*-positive patients with advanced GC had a higher risk of nonclinical response to anti-PD-1 antibody compared to the *H. pylori-negative* group, which also showed prolonged progression-free survival and overall survival [99].

A study on patients with GI advanced cancer treated with PD-1/PD-L1 showed that responders to immunotherapy harbored a relative abundance of *Prevotella* and *Bacteroides* [100]. Another study individuated the trend of abundance of *Rumonococcus faecis* as a possible predictor for distinguishing patients with progressive disease from those with non-progressive disease in all patients with gastrointestinal cancer [7]. Data on the role of microbiota in response to immunotherapy in GC need further investigation. Table 1 cites the original studies assessing the role of gut microbiota in gastric cancer and response to immunotherapy.

## 5. Gut Microbiota, Nutrition, and Related Immunotherapy Outcomes

### 5.1. Gut Microbiota, Diet, and Immunotherapy Response

Several studies have demonstrated the direct and strong influence of diet on the composition of the microbiota [102]. It has been demonstrated that the ratio between Bacteroidetes and Firmicutes, the main phyla of gut microbiota, plays an important role in the pathogenesis of gastrointestinal diseases, and diet is directly involved in changes in microbiota compositions and alterations of this ratio, causing dysbiosis [104].

The Western diet, typically constituted by high consumption of animal fat, high sugar foods, and low amounts of fiber, causes an increase in Firmicutes, Bacteroides, and Enterobacteriaceae (such as *Escherichia coli*, *Klebsiella*, and *Shigella*) and decreases the number of beneficial bacteria (*Bacteroidetes*, *Prevotella*, and *Lactobacillus*) [105]. Recent studies showed that the Western diet can cause dysbiosis by increasing lipopolysaccharides (LPS, a proinflammatory molecule produced by some Gram-negative bacteria), trimethylamine-N-oxide (TMAO), decreasing SCFAs, and increasing the expression of claudin-1 and occludin. These events, associated with altered permeability of the gut barrier, known as leaky gut, are important triggers for low-grade systemic inflammation [106]. Indeed, this abnormal permeability results in LPS translocation, which activates Toll-like receptor 4 (TLR4, represented on most cells and also on macrophages), which, in turn, recognizes pathogen-associated molecular patterns (PAMP). The binding of LPS to TLR4 causes the release of many cytokines that induce an inflammatory response [107].

**Figure 1:**
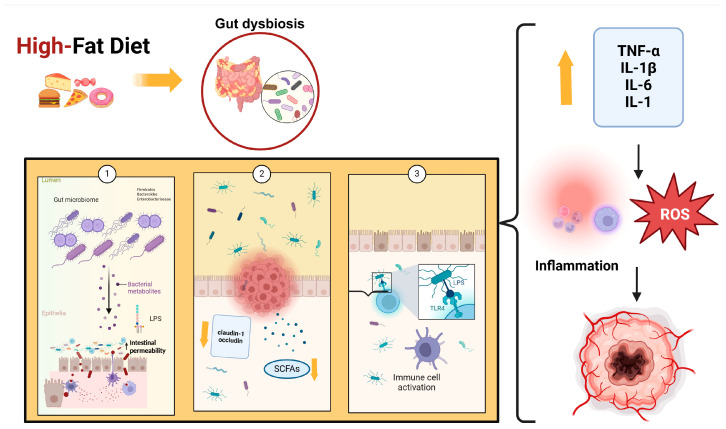
High-fat diet leads to an increase in Firmicutes, Bacteroides, and Enterobacteriaceae, which cause dysbiosis by increasing LPS (1), decreasing SCFAs, and increasing the expression of claudin-1 and occludin (2). These events, associated with altered permeability of the gut barrier, are important triggers for LPS translocation, which activates TLR4 (3). The binding of LPS to TLR4 causes the release of many cytokines and ROS that induce the inflammatory response. Abbreviations: LPS: lipopolysaccharides; SFCAs: short-chain fatty acids; TLR4: Toll-like receptor 4; ROS: Reactive Oxygen Species; TNF-α: Tumor Necrosis Factor-alpha; IL-1β: Interleukin 1-beta; IL-6: Interleukin-6; IL-1: Interleukin 1.

Several studies have also demonstrated that a high-fat diet reduces SCFAs, which are important because they regulate energy metabolism and are involved in immune system modulation through different mechanisms, such as stimulating mucin synthesis, increasing tight junctions’ expression, and inhibiting the NF-kB stimulation pathway [108]. Some bacteria, such as *Akkermansia*, *Lactobacillus*, and *Bifidobacterium*, can increase SCFA production. Specifically, *Bifidobacteria* can protect the integrity of the intestinal barrier and promote the production of anti-inflammatory cytokines and antioxidant products. It has been demonstrated that other proinflammatory effects of a high-fat diet are due to the high content of saturated fatty acids. These products, like LPS, can activate TLR4, release proinflammatory cytokines, impair gut barrier function, and change cellular metabolism. Fat overconsumption leads to mitochondrial oxidation of free fatty acids, increasing ROS production, which can cause a proinflammatory response [109]. Figure 1 illustrates the role of a high-fat diet in the etiopathogenesis of cancer.

The Mediterranean diet is characterized by a high amount of fiber, flavonoids, polyphenols, unsaturated fats, and a low content of animal protein and saturated fats. Dietary fiber plays a prebiotic role in the microbial growth of bacteria [110]. Fibers’ intake is associated with an increased abundance of potentially beneficial species, such as *Akkermansia municiphilia* and *Roseburia* spp., which can produce SCFAs that are important for maintaining epithelial barrier function and regulating the immune system [111]. Fibers can influence the immune system because they stimulate the synthesis of a set of glycans by the bacteria of the Bacteroidetes phylum. Some commensal bacteria, such as *B. fragilis*, can produce an immunosuppressive glycan, the polysaccharide A, which acts like a toll-like receptor 2 (TLR2) ligand and can promote T-regs’ differentiation [112]. Furthermore, polyunsaturated fatty acids (PUFA), such as linolenic acid (n-3 PUFA) and linolenic acid (n-6 PUFA), can modulate the inflammatory response and reduce oxidative stress by inducing an increase in bacteria-producing SCFAs. They can also interact with the G protein-coupled receptor 120 (GPR120), which is expressed by macrophages, reducing the production of tumor necrosis factor-alpha (TNF-α) and Il-6. Vegetables, fruits, and legumes are full of flavonoids. Their uptake is positive for SCFA production and LPS reduction, and they increase *Bifidobacterium* and *Lactobacillus* in the gut microbiota and decrease Enterobacteriaceae [113]. The Mediterranean diet is associated with beneficial changes because it increases the total amount of bacteria and improves the biodiversity of the gut microbiota. Several studies have shown that it increases levels of *Lactobacillus* and *Prevotella* compared to the Western diet, and in patients with high adherence, it promotes the growth of short-chain fatty acids, produces Bacteroidetes, and limits Firmicutes development [114].

Conventional modalities of treatment, such as chemotherapy and radiotherapy, have been used for their cytotoxic effects on cancer cells but, unfortunately, can cause many side effects and damage to healthy tissue. Since immunotherapy has played a key role in cancer treatment in recent years, it has become the first-line treatment for many malignancies, including lung cancer, melanoma, and genitourinary cancers [115]. Therefore, it has been shown that different types of diet can modulate the gut microbiota and, thanks to this effect, can influence the efficiency of immunotherapy [116]. The best strategies to enhance the positive effect of immunotherapy are dietary modifications, avoiding inappropriate use of antibiotics, and correct administration of prebiotics and probiotics [117]. Several studies have proven that individuals with obesity have an increased risk of developing cancer due to their proinflammatory state and suppression of immune system functions [118]. In these patients, obesity leads to the predominance of Firmicutes over Bacteroidetes, negatively affecting immunotherapy outcomes. Indeed, data show that weight loss leads to higher Bacteroidetes levels, and calorie restriction can also increase the effectiveness of immunotherapy [119].

Recent work has revealed that a caloric restriction diet and intake of fermentable oligosaccharides, disaccharides, monosaccharides, and polyols are associated with an enhancement of *Akkermansia muciniphila* (*A. muciniphila*) in the gut microbiota, which improves the response to immunotherapy [120]. Several analyses have demonstrated the influence of many dietary factors on the immune system and their effect on immunotherapy. Fiber intake can modulate the production of SCFAs and boost the presence of beneficial species, such as *Faecalibacterium prausnitzii* and *Roseburia* spp. [121]. Vitamin D can enhance the efficacy of immune checkpoint inhibitor therapy. It exerts a direct effect, inhibiting the Th1 and Th17 cell responses and decreasing inflammatory cytokine synthesis [122]. Studies have proven that this vitamin may increase PD-1 and CTLA-4 expression in T cells and promote Treg cell development [123]. Over the years, many agents, such as prebiotic and probiotic agents, have been studied to understand how to modulate the gut microbiota.

**Figure 2:**
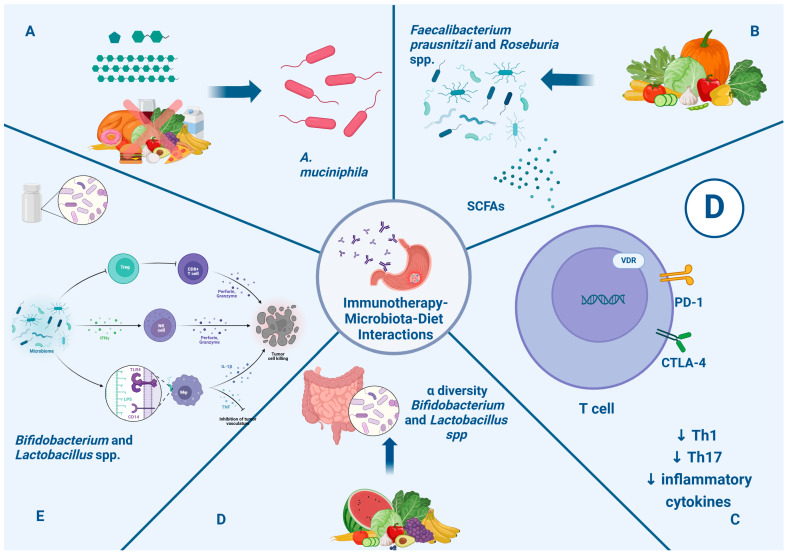
Impact of nutritional intervention through microbiota in immunotherapy in gastric cancer. Section (**A**): Caloric restriction and intake of fermentable oligosaccharides, disaccharides, and monosaccharides are associated with enhancement of *A. muciniphila*, which is associated with a positive systemic effect on immune homeostasis and favorable outcome checkpoint blockade in cancer immunotherapy. Section (**B**): Fiber intakes enhance the presence of *Faecalibacterium prausnitzii* and *Roseburia* spp. and the metabolism of SCFAs, which can promote a clinical response to immunotherapy. Section (**C**): Vitamin D can modulate and favor homeostasis of the immune system through the regulation of PD-1 and CTLA-4 expression and reduction in Th1, Th17, and inflammatory cytokines, with a positive impact on immunotherapy response. Section (**D**): Prebiotics improve alpha diversity and increase the abundance of *Bifidobacterium* and *Lactobacillus* in the gut microbiota, enhancing immunomodulatory effects. Section (**E**): Probiotics like *Bifidobacterium* and *Lactobacillus* spp. can enhance the production of INFγ, which can stimulate NK cells; moreover, LPS-TLR4 binding induces macrophages and the production of inflammatory cytokines. Finally, they lead to enhanced CD8+ T cell priming and accumulation in the tumor microenvironment, promoting antitumor immunity. Abbreviations: CTLA-4: anti-cytotoxic T-lymphocyte antigen 4; anti-PD-1: anti-programmed death cell-1; IFN γ: interferon gamma; IL-1β: interleukin beta; LPS: lipopolysaccharides; NK: natural killer; SCFAs: short-chain fatty acids; TLR4: Toll-like receptor 4; TNF: tumor necrosis factor.

Prebiotics are non-digestible food ingredients like inulin, oligofructose, fructo-oligosaccharides, and galacto-oligosaccharides that can improve alpha-diversity and increase the abundance of *Bifidobacterium* and *Lactobacillus* in the gut microbiota, enhancing their immunomodulatory effects [124,125]. Probiotic supplements are made of live microorganisms like *Bifidobacterium* and *Lactobacillus* spp. They can regulate the host’s immune response and modulate the immune response network. Many trials have shown that the use of probiotics can restore intestinal flora and reduce the side effects of cancer therapies, such as diarrhea [126]. Furthermore, some studies showed that treatment with probiotics enhanced the immunotherapy response [127]. Figure 2 illustrates the impact of nutritional intervention through the microbiota in immunotherapy for gastric cancer.

### 5.2. Gut Microbiota, GC Patient Nutritional Status, and Immunotherapy Response

#### 5.2.1. Associations of Patient Nutritional Status with Immunotherapy Outcomes

In the treatment of immune checkpoint inhibitors, it is of primary importance to assess certain body composition parameters that may be predictive of a better or worse response to treatment. Various studies in the literature have focused on the correlation between body mass index (BMI) and obesity with ICI therapy for solid tumors and, in some specific cases, for GC. It is generally known that a higher BMI improves treatment response. Specifically, obese patients develop a meta-inflammation that results in the secretion of proinflammatory molecules, which seems to increase progression-free survival in this patient group. This phenomenon is known as the “obesity paradox” [93,128,129].

Another parameter that has been evaluated is the skeletal muscle index (SMI), which is indicative of muscle mass. Patients with a high SMI are more likely to survive following ICI administration. This is because muscles contain a certain amount of water, and since antibodies are hydrophilic molecules, they are more likely to be effective in patients with higher muscle mass. A low SMI value is observed in malnourished patients who, due to inflammation, experience muscle degradation [130]. Also related to muscle mass, the psoas muscle index (PMI) can be considered. A low PMI value is thought to indicate a shorter progression-free survival [131]. It is known that sarcopenia, characterized by a loss of muscle mass, is predictive of worse treatment outcomes. The reduction in muscle mass, leading to a decrease in myokine production, results in a reduced modulation of the response to immunotherapy [129].

Other studies have focused on the role of the Prognostic Nutritional Index (PNI) as a predictor of response to ICI therapy. This value quantifies the serum albumin count and lymphocyte count in peripheral blood. A high PNI observed, especially in young and physically fit patients, indicates better overall survival (OS) and progression-free survival (PFS). Malnutrition is associated with an increase in catabolic activity, reflected in a greater elimination of antibodies that undergo catabolic degradation [132]. In conjunction with PNI, the neutrophil-to-lymphocyte ratio (NLR) can also be assessed. In this case, a high pretreatment value is indicative of an increase in the adverse effects of therapy [133,134].

Finally, the controlling nutritional status (CONUT) can be considered a predictor of response to therapy. This parameter considers lymphocytes (indicators of immune response), cholesterol, and albumin (indicators of nutritional status). Lymphocytes can modulate the immune response, hindering tumor growth; a decrease in lymphocytes leads to a higher CONUT score. A correlation has been observed between a lower CONUT score and better OS and PFS [135].

#### 5.2.2. Associations of Gut Microbiota Composition with Patient Nutritional Status

Researchers have demonstrated that subjects with a lower BMI than obese subjects have a significantly higher abundance of *F. prausnitzii* [136,137]. On the other hand, other bacteria such as *Collinsella* [137,138], *Veillonellaceae* [139], and *Lachnospiraceae* families are found in significantly higher amounts in subjects > 25 kg/m^2^ compared to subjects with typical BMI [137,138,140,141].

The gut microbiota could also be associated with muscle mass. Indeed, in germ-free mice, after transplantation, muscle atrophy and protein synthesis increase compared with germ-free mice [142]. In humans, higher muscle mass is associated with a gut microbiome enriched in *Akkermansiaceae* [143,144], including *A. muciniphila* [145]. The abundance of members of the genus *Faecalibacterium* may also be a marker of higher skeletal muscle mass, as well as *Coprococcus* and *Lachnospiraceae* [146]. In addition, an increase in lean body mass and a relative abundance of *Faecalibacterium* are observed in normal-weight subjects after exercise training [138]. On the contrary, in elderly subjects affected by frailty and sarcopenia, a lower abundance of *F. prausnitzii*, *Clostridiales*, and *Roseburia* was observed compared to controls [147,148]. In animal studies, *A. muciniphila* abundance is negatively associated with adipose tissue [149,150]. On the other hand, *Coprococcus* abundance correlates positively with the level of subcutaneous body fat [151]. Thus, the gut microbiota could be different according to the amount of fat or muscle mass. Further studies are needed to explain these differences in terms of quantity and composition. In GC, it is well known that muscle mass is associated with improved treatment response and overall survival [152]. Thus, the gut microbiota profile could be a prognostic marker associating the body composition of GC patients with immunotherapy responses.

## 6. Conclusions

The potential interactions between nutrition, microbiota, and immunotherapy outcomes in GC patients are growing in interest. It is widely known that the gut microbiota plays an essential role in immune responses. The effects of perigastric/gut microbiota compositions/functions and their derivated metabolites could be predictive of response to immunotherapy in GC patients and even PFS and overall survival. At the same time, the strong influence of diet on the composition of the microbiota could have consequences on immunotherapy responses through the impact of the nutritional status, in particular muscle mass, in GC patients during immunotherapy treatment. Studies are needed to elucidate how the interactions between microbiota-dietary interventions and nutritional status could improve treatment response and the overall survival of GC patients.

## Figures and Tables

**Table 1:** Studies assessing the role of gut microbiota in gastric cancer and immunotherapy outcomes. Abbreviations: anti-CTLA-4: anti-cytotoxic T-lymphocyte antigen 4; anti-PD-1: anti-programmed death cell-1; PD-L1: anti-programmed cell death ligand-1; GC: gastric cancer; GI: gastrointestinal; irAEs: immune-related adverse events; n/a: not applicable; PFS: progression-free survival; OS: overall survival; ↑: increase; ↓:decrease.

First Author, Year of Publication	Study Design	Bacteria spp.	Treatment	Outcomes
Che et al., 2022 [101]	Retrospective, single-center	*Helicobacter pylori*	anti-PD-1	↓OS, PFS
Magahis et al., 2023 [101]	Retrospective, single-center	*Helicobacter pylori*	anti-PD-1/PD-L1, anti-CTLA-4 ± chemotherapy	↓OS, PFS
Peng et al., 2020 [7]	Prospective observational	*Prevotella/Bacteroides*, ↑*Prevotella*, *Ruminococcaceae*, *and Lachnospiraceae*	anti-PD-1/PD-L1	↑Response to treatment
Li et al., 2021 [102]	Prospective, observational	*Ruminococcus faecis*	chemotherapy	Progression disease
Liu et al., 2021 [103]	Prospective, observational	*Streptococcus*, *Paecalibacterium*, and *Stenotrophomonas* versus *Faecalibacterium* and *unidentified_Lachnospiraceae*	anti-PD-1 chemotherapy ± antiangiogenic	Severe irAEs vs. mild irAEs

## Data Availability

Not applicable.
